# Guideline-based stepped and collaborative care for patients with depression in a cluster-randomised trial

**DOI:** 10.1038/s41598-018-27470-6

**Published:** 2018-06-20

**Authors:** Martin Härter, Birgit Watzke, Anne Daubmann, Karl Wegscheider, Hans-Helmut König, Christian Brettschneider, Sarah Liebherz, Daniela Heddaeus, Maya Steinmann

**Affiliations:** 1 Department of Medical Psychology, University Medical Center Hamburg-Eppendorf, Martinistraße 52, 20246 Hamburg, Germany; 2 Clinical Psychology and Psychotherapy Research, Institute of Psychology, University of Zurich, Binzmühlestrasse 14/16, CH-8050 Zurich, Switzerland; 3 Department of Medical Biometry and Epidemiology, University Medical Center Hamburg-Eppendorf, Martinistraße 52, 20246 Hamburg, Germany; 4 Department of Health Economics and Health Services Research, Hamburg Center for Health Economics, University Medical Center Hamburg-Eppendorf, Martinistraße 52, 20246 Hamburg, Germany

## Abstract

Guidelines recommend stepped and collaborative care models (SCM) for depression. We aimed to evaluate the effectiveness of a complex guideline-based SCM for depressed patients. German primary care units were cluster-randomised into intervention (IG) or control group (CG) (3:1 ratio). Adult routine care patients with PHQ-9 ≥ 5 points could participate and received SCM in IG and treatment as usual (TAU) in CG. Primary outcome was change in PHQ-9 from baseline to 12 months (hypothesis: greater reduction in IG). A linear mixed model was calculated with group as fixed effect and practice as random effect, controlling for baseline PHQ-9 (intention-to-treat). 36 primary care units were randomised to IG and 13 to CG. 36 psychotherapists, 6 psychiatrists and 7 clinics participated in SCM. 737 patients were included (IG: n = 569 vs. CG: n = 168); data were available for 60% (IG) and 64% (CG) after 12 months. IG showed 2.4 points greater reduction [95% confidence interval (CI): −3.4 to −1.5, p < 0.001; Cohen’s d = 0.45] (adjusted PHQ-9 mean change). Odds of response [odds ratio: 2.8; 95% CI: 1.6 to 4.7] and remission [odds ratio: 3.2; 95% CI: 1.58 to 6.26] were higher in IG. Guideline-based SCM can improve depression care.

## Introduction

Depression is one of the most widespread mental disorders^1^. Although successfully treatable, depression often remains undetected or is diagnosed late^2,3^ and often treatment is delayed or not evidence-based^3,4^. Treatment selection is often unsystematic regarding type and intensity^5^ and fragmented healthcare services encumber integrated care^6^. Guidelines such as the NICE guideline “The treatment and management of depression in adults (updated edition)”^7^ and the German National Clinical Practice Guideline “Unipolar Depression” (updated edition)^8^ recommend stepped and collaborative care to improve depression care^5,7–10^. Stepped care aims to treat patients with an adequate treatment of the lowest possible intensity while continuously monitoring progress. Stepped care is usually combined with collaborative care, which aims to systematically integrate different care providers^11,12^. Robust evidence confirms the effectiveness and cost-effectiveness of collaborative care for depression^11,13,14^ and results regarding stepped care for depression are promising, although less conclusive^15,16^.

While all stepped care models include care management elements, they vary greatly concerning amount and type of steps, care providers and stepping-up criteria^16^. Several stepped care interventions integrate care management, self-help, low-intensity psychotherapeutic interventions and antidepressant medication systematically into one model e.g.^17^, of which only three were evaluated within randomised controlled trials^18–20^. One preventive model addressed residents in homes for the elderly^18^, one addressed women in low-income community practices in Chile^19^, while the third incorporated care managers into general practitioners’ (GP) practices, thus using additional resources hardly pertaining to regular care^20^.

Furthermore, very few stepped and collaborative care models exist in which inpatient treatment plays more than a marginal role, although this setting is necessary for severely ill patients, especially with chronic depression or suicidality^8^. More randomised controlled trials (RCT) are needed on complex, guideline-based care models with a broad intervention spectrum. It appears especially important to evaluate approaches making efficient use of established health care structures to facilitate later implementation and roll out in routine care, as opposed to models which require bringing external care managers into practices.

The primary objective of this study was to investigate the effectiveness of a stepped and collaborative care model (SCM) for patients with depression based on the German National Clinical Practice Guideline “Unipolar Depression”^8^, incorporating treatment options of various intensities in out- and inpatient settings. We hypothesised that SCM would lead to greater depressive symptom reduction from baseline to 12 months on an individual patient level. A cluster-randomised design was chosen because it was necessary to provide GPs in the SCM condition with specialised training and tools.

This model and the accompanying study were implemented under routine conditions involving care providers active in regular health care. This type of setting contrasts with many other stepped care studies implemented to date, which rely on bringing new care providers into the system. Such approaches would be difficult to implement in countries where no large-scale government initiative supports these projects, such as Germany.

## Methods

### Study design

The study, described in detail elsewhere^21^, was embedded into the research initiative *psychenet - The Hamburg Network for Mental Health*^22^. It was designed as a prospective parallel cluster-randomised controlled intervention trial of a consecutive sample of patients with depression from primary care assessed at four time points. Participating GP practices were randomised to intervention group (IG) or control group (CG) in a 3:1 ratio. The GP practices consented to be randomised before randomisation took place and were not blinded to group assignment. The randomisation process was conducted by a computer program using minimisation based on practice size, practice location and income level of the practice’s local district.

Sample size calculation was based on the detection of a small to moderate effect (Cohen’s d of 0.40) with a statistical power of 0.80 and a type I error rate of 0.05 between each of six treatment options in SCM and TAU. We aimed to recruit three times more patients in IG than in CG in order to run further separate subgroup analyses for each of the six treatment options available in IG. After considering the clustered design with an expected intra-cluster correlation (ICC) of 0.05, the differential expected attrition rates between groups, and the amount of patients needed in IG in order to run the further separate analyses, we aimed to recruit a total of 860 patients. Expecting that each of the GPs in the included practices would recruit 15–25 patients, we planned to gain 40 practices for study participation (660 patients from 30 practices in SCM and 200 patients from 10 practices in TAU^21^). In total, we recruited 49 practices: 36 practices were randomised to the IG and 13 practices to the CG.

Patients in SCM were assessed and treated within an integrated network comprising 36 GP practices, 36 psychotherapists, 6 psychiatrists and 7 inpatient clinics^23,24^, while patients in TAU were assessed and treated in 13 GP practices and any available routine care facilities. No changes were made to methods after trial commencement.

#### Approval

The study was approved by the Ethics Committee of the Hamburg Chamber of Psychotherapists.

#### Accordance

The study was conducted according to the principles of the Declaration of Helsinki (2013 version).

#### Informed consent

Written informed consent was obtained from all participants.

#### Clinical trial registration

The study was registered in ClinicalTrials.gov (registration number: NCT01731717; registration date: 11.12.2012).

### Care Providers

GPs in greater Hamburg were invited to participate by mail via the Hamburg Chamber of Physicians. Inclusion criteria were to be working as a GP in an established GP practice and willingness to participate in study procedures. Once we reached the planned sample size of at least 40 practices (see sample size calculation and results section), we did not follow up on this initial invitation, i.e. did not send out reminder letters.

### Patients

GPs recruited patients in three screening and assessment steps using checklists and the PHQ-9^21^. Inclusion criteria were: minimum age of 18, Patient Health Questionnaire-9 (PHQ-9) score ≥ 5 and informed consent. Exclusion criteria were insufficient German language knowledge or a health situation not permitting questionnaire completion. Neither somatic nor mental comorbidities were exclusion criteria; patients were only excluded if a comorbid mental disorder (e.g. trauma) was the main treatment focus.

### Interventions

#### Stepped collaborative care model (SCM)

SCM was a stratified stepped and collaborative care approach carried out by GPs, psychiatrists, psychotherapists and inpatient facilities in the IG^21^. GPs completed an ICD-10-based checklist and imparted psychoeducation. Treatment interventions were allocated by GPs following guideline recommendations, i.e. based on depression severity and patient preferences (shared decision-making)^21^.

Treatment options on four intensity levels were available: *Step 1*) active monitoring; *2a)* bibliotherapy; *2b)* internet-based self-management; *2c)* telephone-administered psychotherapy (9–13 sessions); *3a)* outpatient psychotherapy in individual or group settings (usually up to 25 sessions); *3b)* antidepressant pharmacotherapy; and *4)* combination of psycho- and pharmacotherapy in out- or inpatient setting.

Depression severity (PHQ-9) was monitored by care providers in predefined intervals following guideline recommendations. Stepping up was recommended if PHQ-9 score did not improve by at least 20%. A care provider network and an online platform indicating available treatment capacities in secondary care were implemented to facilitate communication and referral. All care providers obtained intensive training regarding guideline recommendations^7,8^, psychoeducation, SCM and related interventions. Quarter-yearly quality circles assured SCM quality and adherence to guideline recommendations.

#### Treatment as usual (TAU)

CG patients received treatment as usual by their GP and within regular German healthcare, including potentially necessary referrals to psychotherapy and psychiatry in out- or inpatient facilities. Systematic screening (see “Patients”) was carried out in both SCM and TAU to ensure a comparable recruitment and inclusion process. However, CG care providers did not have access to training and quality circles, diagnostic and decision-making tools, systematic monitoring, low-intensity treatments or online referral tools.

### Outcomes

All outcomes were assessed by self-report questionnaires at four time points: Baseline (T0) assessment was handed out by the GP at study inclusion and completed before treatment began. Patients received further questionnaires 3 (T1), 6 (T2) and 12 months (T3) after baseline by mail. If a questionnaire was not returned within two weeks, up to two reminder letters were sent to the patient and one to the patient’s GP to improve response rates. No changes were made to outcome measures after the trial commenced.

Primary outcome was change in depressive symptoms assessed by the Patient Health Questionnaire-9 (PHQ-9)^25^ from baseline to 12 months. We hypothesised that SCM would reduce depressive symptoms significantly more than TAU from baseline to 12 months. Secondary outcome parameters were change in depressive symptoms over various time points (PHQ-9), response (≥50% reduction on PHQ-9 from T0 to T3), remission (<5 points on PHQ-9 at T3), change in health-related quality of life assessed by the 12-item Short Form Health Survey (SF-12)^26^ and patient satisfaction assessed by the Client Satisfaction Questionnaire (CSQ-8)^27^.

### Statistical analysis

The primary outcome analysis was based on the intention to treat (ITT) population. In case of missing follow-up values, a last-observation carried forward (LOCF) imputation was performed in adherence to our study protocol^21^. Using this data, a linear mixed model was calculated with group (SCM/TAU) as a fixed effect and GP practice as a random effect under control of baseline PHQ-9 score as a covariate. Sensitivity analyses were performed for the primary outcome by applying multiple imputation (MI) as a further method of missing value imputation. The intra-cluster correlation (ICC) was computed to assess the proportion of variance explained by the clusters.

For each secondary outcome parameter, a linear mixed model was computed with group (SCM/TAU) as fixed effect and practice as random effect. For the analysis of change in depressive symptoms over various time points, baseline PHQ-9 score was controlled for as a covariate. For the analysis of change in health-related quality of life, baseline SF-12 score was included as a covariate. Group-by-time interactions were computed. Here, the adjusted means of between-groups differences were only reported if group-by-time interaction was significant. No covariates were included in analyses of remission, response and patient satisfaction. For all analyses, a significance level of p = 0.05 was determined and SPSS 21 was used.

### Data availability statement

The datasets generated and analysed during the current study are available from the corresponding author on reasonable request.

## Results

### Participant flow

**Figure 1:**
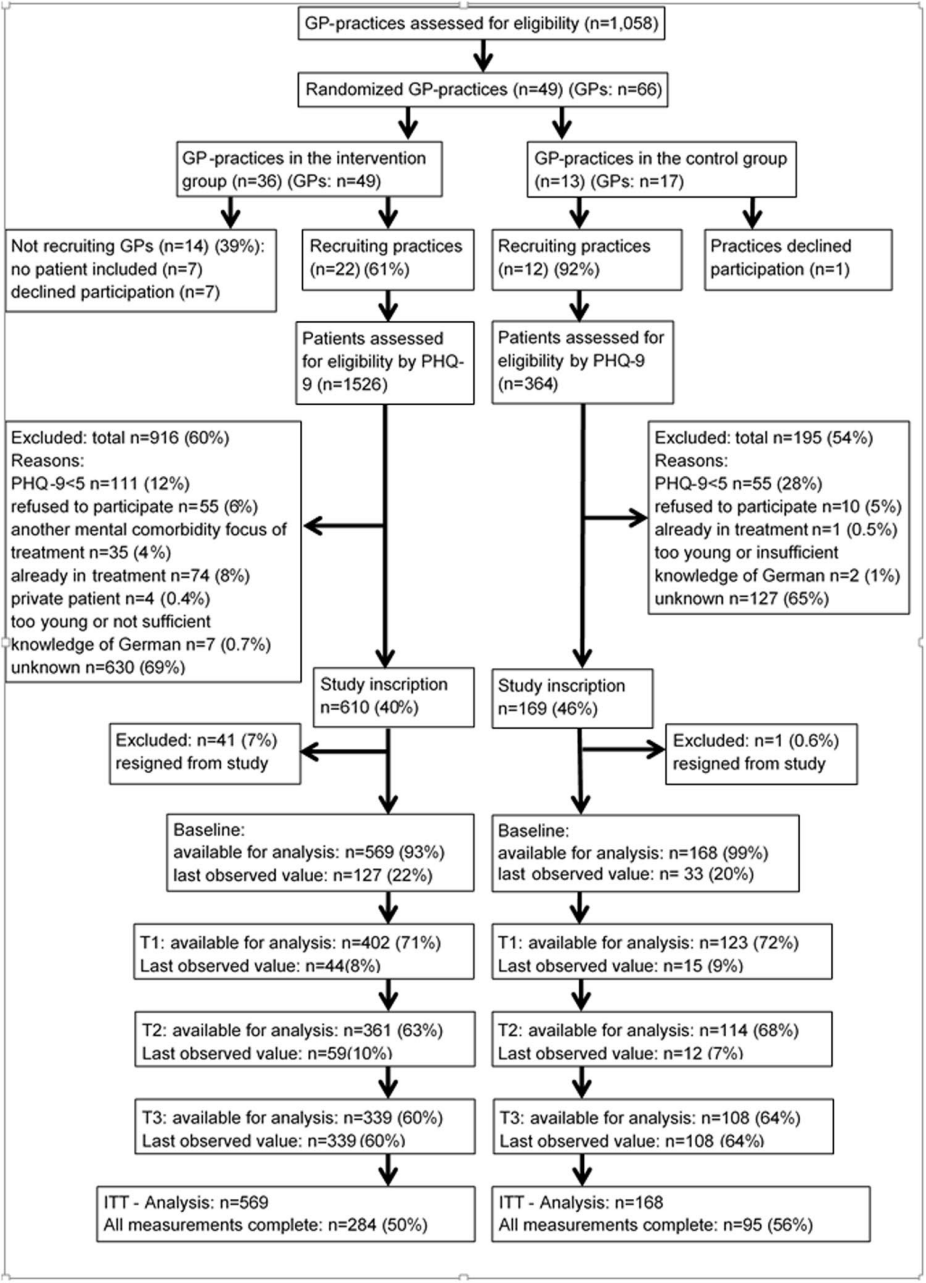
Participant flowchart according to the cluster-randomised CONSORT statement^28^.

Figure 1 shows participant flow according to the extended CONSORT statement for cluster-randomised trials^28^: Between September 2011 and April 2012, 1,058 practices were invited to participate, of which 49 practices finally agreed and were randomised. Of the 36 practices randomised to SCM, 22 actively included at least one patient; in TAU, this was the case in 12 of 13 practices.

We collected data regarding the GP practices in IG who failed to actively recruit patients. These GPs and GPs in active IG practices showed similar characteristics regarding age, years working in an own practice, number of patients treated and proportion of depressive patients. However, GPs failing to recruit patients were more often female (88.9% as opposed to 68.8% in active practices) and worked notably longer mean hours per week (51.1 hours (SD = 12.6 hours) as opposed to 41.1 hours (SD = 11.4 hours) in active practices). We did not have the opportunity to collect systematic data regarding the characteristics of GPs who dropped out directly after randomisation, but were only able to document anecdotal reasons. In most cases, GPs stated time constraints due to the extra documentation workload required in SCM. Other reasons were problems integrating study procedures into practice routine, and the decision to participate in a competing research study, among others.

A total of 1,890 patients were screened for eligibility with PHQ-9, of which 779 patients gave informed consent (610 in IG, 169 in CG). The proportion of patients who didn’t fill out questionnaires was comparable in SCM and TAU at all time points. Patients with missing T3 questionnaires didn’t differ significantly from patients who filled out the questionnaire regarding baseline depression severity (PHQ-9), partnership status, education level or nationality. However, patients with missing T3 questionnaires were significantly younger (mean age_missing_ =  39.6 (SD = 13.9); mean age_not missing_ =  45.0 (SD = 13.7); p < 0.001) and more often male (female = 36% missing vs. male = 49% missing; p = 0.001).

### Sample

Patient inclusion took place from August 08, 2012 to March 31, 2014. Follow-up measurements took place between 2012 and 2015. SCM and TAU do not appear to display relevant differences regarding baseline characteristics of individuals on patient or cluster level (Table 1). Approximately three of four included patients were female, with a mean age in the early to mid-forties.

**Table 1:** Baseline characteristics on patient and cluster level in SCM_a_ and TAU_b_.

Patient level	Group	
	SCM_a_ (n = 569)	TAU_b_ (n = 168)
Age (M (SD))	42.1 (13.5)	45.6 (15.5)
Female gender (%)	72.8	75.6
Education level (%)		
*Secondary general school*_c_	20.4	29.8
*Intermediate secondary school*_d_	27.6	26.2
*High school*_e_	25.1	22.0
*University or technical college degree*	14.2	10.1
*No school degree*	2.1	3.0
Employment status (%)		
*No employment*	29.1	34.7
*Minor employment*	3.5	4.1
*Part-time employment*	20.9	18.4
*Full-time employment*	46.5	42.9
Living in partnership (%)	54.1	52.4
PHQ-9 at baseline (M (SD))	15.3 (4.7)	14.1 (4.9)
**Cluster level (GPs)**	**SCM**_**a**_ **(n** = **36)**	**TAU**_**b**_ **(n** = **13)**
Age (M (SD))	49.6 (7.0)	50.1 (9.4)
Female gender (%)	68.8	57.1
Working in own practice (years (SD))	13.5 (7.7)	10.4 (7.8)

_a_SCM = Stepped collaborative care model (intervention group); _b_TAU = Treatment as usual (control group).

_c_German: Hauptschule (9 years of education); _d_German: Realschule (10 years); _e_German: Gymnasium (13 years).

### Primary outcome

**Figure 2:**
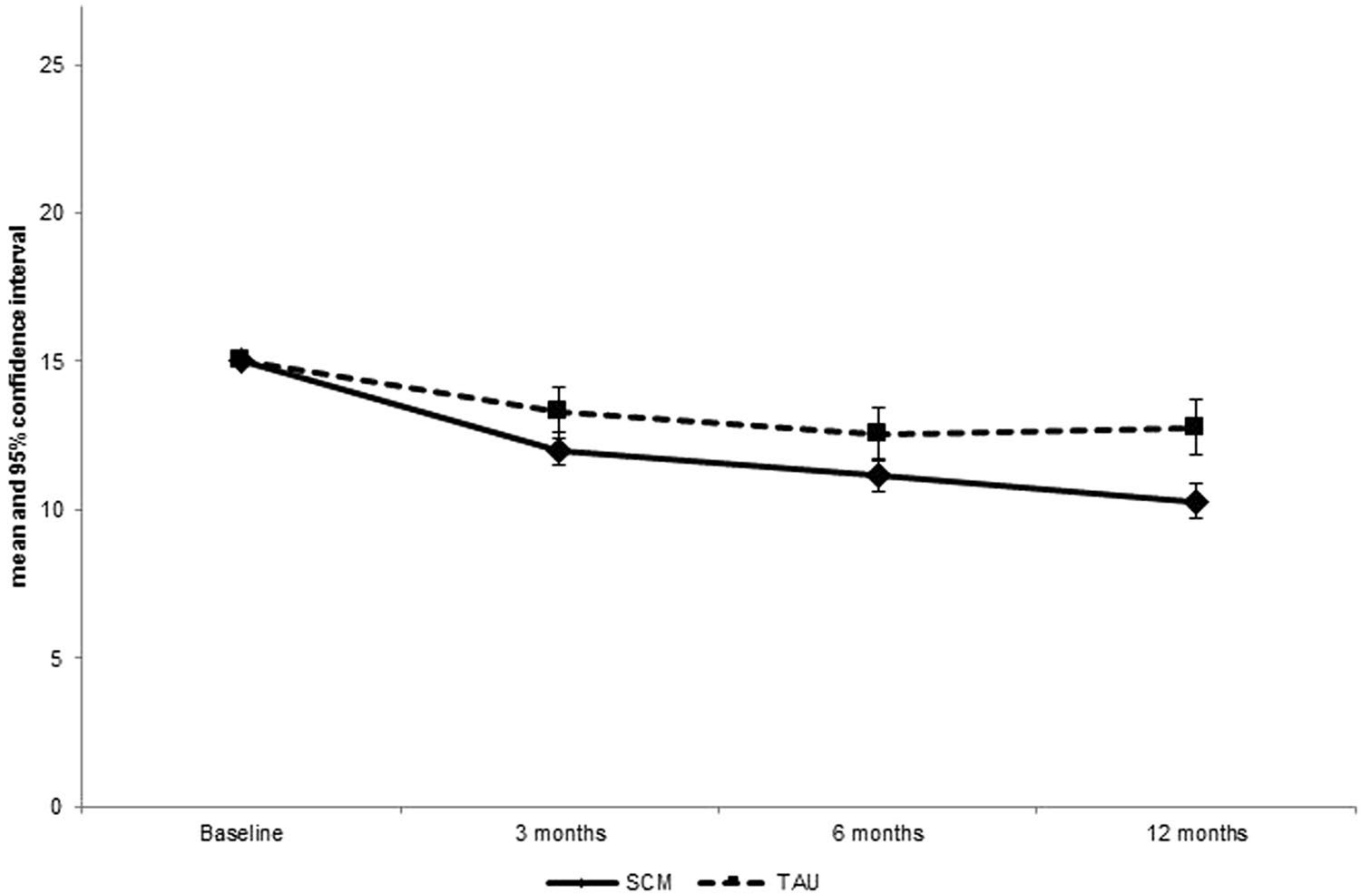
Adjusted PHQ-9 scores over time in SCM and TAU.

The adjusted PHQ-9 mean reduction from baseline to 12 months was 2.44 points greater in patients in SCM than in TAU [95% CI: −3.4 to −1.5, p < 0.001; Cohen’s d = 0.45] (Fig. 2, Table 2). Sensitivity analysis performed by imputing missing values with MI instead of LOCF yielded a very similar and significant 2.53-point difference between SCM and TAU [95% CI: −3.5 to −1.6, p < 0.001; Cohen’s d = 0.47]. An intra-cluster correlation (ICC) of 0.0032 was found, meaning that only 0.3% of variation in depressive symptoms (PHQ-9) at 12 months could be attributed to GP practices after taking baseline depressive symptom severity of patients and treatment group membership of practices into consideration.

**Table 2:** Primary and secondary outcome assessments for patients in SCM and TAU.

Outcome	SCM_a_ n = 569		TAU_b_ n = 168		Between groups difference: Adjusted mean (95% CI)	Between groups difference: p-value	Group x time interaction	Effect size (Cohen’s d)
	Mean (SD)	Adjusted mean change (95% CI)	Mean (SD)	Adjusted mean change (95% CI)				
**Primary outcome**								
***Depression severity from T0 to T3 (assessed by PHQ-9***_**c**_ ***, LOCF***_d_ ***)***								
Baseline	15.29 (4.68)		14.09 (4.92)			<0.0001	—	0.45
12 months	10.33 (6.03)	−4.80 (−5.31; −4.29)	12.12 (5.53)	−2.36 (−3.20; −1.52)	−2.44 (−3.42; −1.46)			
***Depression severity from T0 to T3*** ***(assessed by PHQ-9, MI***_**e**_ *)*								
12 months	8.91 (5.50)	−6.20 (−6.77; −5.62)	10.98 (5.50)	−3.67 (−4.63; −2.71)	−2.53 (−3.49; −1.56)	< 0.0001	—	0.47
**Secondary outcomes**								
***Depression severity over all time points*** *** (assessed by PHQ-9, LOCF)***								
Baseline	15.29 (4.68)		14.09 (4.92)				0.003	
3 months	12.06 (5.81)	−3.00 (−3.54; −2.47)	12.62 (5.33)	−1.74 (−2.61; −0.87)	−1.26 (−2.29; −0.24)	0.016		0.23
6 months	11.21 (5.92)	−3.85 (−4.40; −3.30)	11.90 (5.37)	−2.46 (−3.37; −1.55)	−1.39 (−2.46; −0.33)	0.011		0.24
12 months	10.33 (6.03)	−4.73 (−5.30; −4.16)	12.12 (5.53)	−2.24 (−3.18; −1.30)	−2.49 (−3.59; −1.39)	<0.0001		0.41
***Mental health over all time points*** *** (assessed by SF-12***_**f**_ ***, LOCF)***								
Baseline	28.41 (8.33)		30.56 (9.28)				0.093	0.13
3 months	33.45 (11.16)	4.56 (3.45; 5.68)	33.81 (10.37)	2.94 (1.29; 4.60)	1.62 (−0.32; 3.57)	0.098		
6 months	35.22 (11.85)	6.24 (5.07; 7.40)	34.96 (10.49)	4.61 (2.93; 6.30)				
12 months	37.68 (12.66)	8.26 (7.05; 9.46)	35.74 (10.68)	6.63 (4.93; 8.35)				
***Physical health over all time points*** *** (assessed by SF-12, LOCF)***								
Baseline	44.64 (10.59)		42.03 (10.45)				0.043	
3 months	45.26 (9.99)	0.75 (−0.42; 1.54)	42.32 (10.85)	−0.27 (−1.50; 0.96)	1.02 (−0.44; 2.48)	0.166		0.13
6 months	46.15 (9.99)	1.60 (0.80; 2.41)	42.46 (11.52)	−0.180 (−1.45; 1.09)	1.78 (0.28; 3.28)	0.021		0.22
12 months	46.38 (9.99)	1.838 (0.98; 2.70)	41.66 (10.91)	−1.08 (−2.46; 0.30)	2.92 (1.30; 4.54)	0.001		0.33
***Client satisfaction at T3*** *** (assessed by CSQ-8***_**g**_ ***)***								
12 months	25.19 (5.25)		24.23 (4.38)		0.96 (−0.33; 2.26)	0.138	—	0.15
***Response and Remission***								
	**SCM**		**TAU**		**SCM vs. TAU**			
	**n**	**%**	**n**	**%**	**OR**	**95% CI**	**p**	
Response_h_	196	34.5%	25	14.9%	2.8	1.63–4.74	0.001	
Remission_i_	115	20.2%	12	7.1%	3.2	1.58–6.26	0.001	

_a_SCM = Stepped collaborative care model (intervention group).

_b_TAU = Treatment as usual (control group).

_c_PHQ-9 = Patient Health Questionnaire.

_d_LOCF= Last observation carried forward.

_e_MI = Multiple imputation.

_f_SF-12 = Short form questionnaire.

_g_Client satisfaction questionnaire.

_h_Response = at least 50% reduction on the PHQ-9 from baseline to 12 months.

_i_Remission = a PHQ-9 score below 5 points at 12 months.

### Secondary outcomes

34.5% (196 of 569) of patients in SCM showed response (at least 50% reduction on PHQ-9 from baseline to 12 months), while this was the case for 14.9% (25 of 168) of patients in TAU. The odds of response were 2.8 times higher in SCM than TAU [95%-CI: 1.63 to 4.74; p < 0.001].

20.2% (115 of 569) of SCM patients achieved remission (PHQ-9 score below 5 points at 12 months), this was the case for 7.1% (12 of 168) of TAU patients. The odds of obtaining remission were 3.2 times higher in SCM [95%-CI: 1.58 to 6.26; p = 0.001].

The analysis regarding change in adjusted PHQ-9 values over various time points demonstrated that depressive symptoms decreased significantly more in SCM than in TAU at all measurement points (1.3 points more at 3 months, 1.4 points more at 6 months, and 2.5 points more at 12 months) (Table 2). Time and treatment allocation interacted significantly.

The SF-12 mental health score didn’t improve significantly more in SCM than in TAU over all time points, while the SF-12 physical health score improved significantly more in SCM at both 6 and 12 months (1.8 and 2.9 points, respectively) (Table 2). Only the physical health subscale of the SF-12 showed a significant interaction between time and treatment allocation.

There was no significant difference regarding patient satisfaction according to CSQ-8 at 12 months between SCM and TAU (Table 2). No adverse events or side effects were reported in SCM or TAU.

## Discussion

This randomised controlled trial demonstrates the effectiveness of a guideline-based stepped and collaborative care model (SCM) for routine depression care. SCM patients showed a significantly greater reduction in depressive symptoms and higher response and remission rates than TAU patients. The almost moderate effect size of d = 0.45 is slightly higher than the effect sizes of d = 0.41 and d = 0.34 found in two reviews on stepped care^15,16^. The 12-month response and remission rates also appear comparable to those found elsewhere^15,20^. The SF-12 mental health score did not improve significantly more in SCM than in TAU, which could be due to the smaller sensitivity to change of this generic instrument. The physical subscale of the SF-12 improved slightly more in SCM; however, we do not consider this to be a clinically relevant difference. There were no significant differences regarding patient satisfaction, which reflects an equally high perceived treatment quality in German routine care. The very low intra-cluster correlation shows that only a negligible amount of variance in effectiveness is explained by being included into the study by a certain GP.

SCM included a wider variety of interventions than most stepped care studies in other RCTs to date, addressed a broad spectrum of patients and was carried out by a comprehensive network of more than 80 care providers, including GPs, psychiatrists, psychotherapists and hospitals. The complex and effective treatments offered were implemented by linking and training routine care providers, without introducing new resources such as care managers or nurses into established practices, as was done in other models, e.g.^20^. Diagnostic checklists, low-intensity interventions and technological tools were implemented successfully and were associated with high care provider satisfaction^24^.

We analysed an ITT sample and imputed missing values by last observation carried forward^21^. The results remained nearly identical when we reanalysed primary outcome using multiple imputation. The patients in our study can be considered representative for patients with mild to severe depression in routine primary healthcare. Thus, results of this trial are more generalizable to this setting than studies focusing on preventive models^18^.

Patients included into SCM and TAU were comparable, which is probably due to the rather large amount of GP practices recruiting patients and the identical inclusion processes. The fact that SCM outperformed TAU remains especially noteworthy considering the high quality of regular German healthcare, since TAU patients had access to specialist care offers.

We stopped recruiting GPs once we had reached a sufficient number of practices. GPs who responded to our invitation had a similar age (approx. 50 years) as the mean of Hamburg GPs in 2012 (M = 53.9 years); however, a higher proportion of study GPs was female (approx. 60–70%, as opposed to M = 40.6% of Hamburg GPs in 2012)^29^. It is a major limitation that only a small proportion of the care providers invited to participate in the study by mail actually took part. This mirrors the experience of other German studies that it is often difficult to motivate GP samples to participate in research: for instance, a study evaluating nurse-led collaborative care reports facing enormous difficulties recruiting only ten GP practices, requiring up to five telephone calls in order to motivate them^30^. The low number of recruited practices could represent a bias in the sense that GPs interested in participating may be more sensitised towards depression and motivated than the mean of GPs. While this is likely to be true of GPs both in SCM and in TAU and thus does not compromise between-group comparability, it could limit external validity and generalizability.

Another limitation is that the drop-out rate of participating GPs in SCM was higher than in TAU, most likely due to the greater efforts requested from GPs in SCM. When comparing participating GPs with those who failed to recruit patients, we found them to have similar characteristics, although GPs failing to recruit were more often female and worked notably more hours per week. The latter seems to confirm the statements made by GPs, who cited time constraints as the most common reason for not actively participating in the study. As we were not able to collect more comprehensive data on these GPs or any systematic data on the GPs who dropped out directly after randomisation, we cannot exclude the possibility that GPs who actively participated were selectively more motivated and/or competent than those who dropped out.

It will be important to further investigate manners of motivating GPs and other care providers to become and remain involved in guideline-based care models^31^. This can be addressed on a micro-level by further developing instruments facilitating diagnostics and monitoring, such as web-based referral tools^23^ and decision aids^20^. Implementation could also be improved by sensitising and training care providers regarding guideline recommendations and financing new integrated care models.

In order to attain more information regarding the implementation of our model, we performed a detailed analysis of which interventions were recommended and actually carried out within the stepped care network, which we reported elsewhere^32^. We are also currently evaluating the study’s cost-effectiveness. Additionally, while a 12-month measurement represents a mid-term outcome, we are currently conducting a three year post-randomisation follow up assessment of the study’s patients as long-term outcome.

We were not able to blind patients to study condition. In order to assure a comparable patient recruitment process, GPs in TAU were instructed to perform the same screening procedures as GPs in SCM. Due to this, detection rates in CG were probably higher than in regular primary care, which is likely to be related to higher treatment rates as well. Finding differences between IG and CG under these conservative preconditions was less likely and the demonstrated effectiveness appears especially robust.

Although all study patients demonstrated elevated levels of depressive symptoms, not all may have met diagnostic criteria for a clinical depression according to ICD-10, as inclusion criterion was PHQ-9-score. While a relatively high percentage of patients failed to fill out all questionnaires, few systematic differences between patients with complete and incomplete data sets were found. These dropout rates appear comparable to those found in many other stepped care models and reflect normal rates in routine care e.g.^20,33^. While some studies e.g.^17^ achieved lower dropout rates, this seems to have been due to more favourable organisational conditions (e.g. screening questionnaires integrated into routine care or the use of data from electronic medical records). The drop-out rate of participating GPs in SCM was higher than in TAU, probably due to the greater efforts requested in SCM.

This RCT shows that guideline-based SCM involving routine care providers leads to a greater reduction of depression scores, to higher response and remission rates than treatment as usual. It is possible to enhance the quality of depression care through guideline-based training, collaborative networks and the use of innovative intervention elements within stepped care approaches. We believe that this study under real-world conditions can have an important impact on health service provision, offering results which could inform roll-out and policy. However, the generalizability of these findings has the major limitations of a very low rate of participation by eligible practices and potential bias caused by the high rate of practice withdrawal in the intervention group. The selection processes and the representativeness of participating care providers in different health care systems should be taken into special account in future studies. Further studies should also investigate the effectiveness of SCM for other prevalent mental disorders and for comorbid mental conditions in primary care.
